# Discovering CO_2_–Reactive Carbanions via Property-Guided Generative AI

**DOI:** 10.1021/acs.jcim.6c00680

**Published:** 2026-06-10

**Authors:** Bo Li, De-en Jiang

**Affiliations:** Department of Chemical and Biomolecular Engineering, 5718 Vanderbilt University, Nashville, Tennessee 37235, United States

## Abstract

Designing carbanions capable of efficient CO_2_ chemisorption is an important approach for advancing reactive capture and conversion technologies. In this work, we integrate a nucleophilicity prediction model, trained using directed message-passing neural networks on Mayr’s Reactivity Database (a large, experimentally derived collection of nucleophilicity, electrophilicity, and sensitivity parameters for organic and inorganic molecules), with the Hierarchical Variational Autoencoder (HierVAE) to generate novel carbanions. Fine-tuning the pretrained latent space with predicted log *k* values yields structurally diverse carbanions with strong CO_2_ reactivity and high generation success. Analysis of the top candidates reveals consistent trends in α-substitution, electron-withdrawing groups, and synthetic accessibility. Density functional theory (DFT) validation of the highest-ranked reactive candidates reveals good agreement for electronically stabilized systems bearing strong electron-withdrawing substituents, while deviations increase for weakly stabilized or sterically distinct carbanions, defining a clear domain of applicability for the predictive model. This study demonstrates the promise of property-guided generative AI for discovering novel carbanions for room-temperature CO_2_ chemisorption.

## Graphical abstract

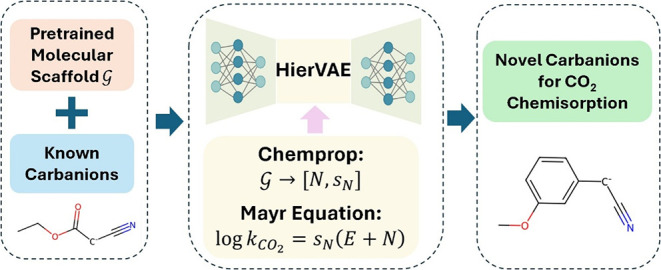

## 1 Introduction

CO_2_ capture and conversion are central scientific challenges in mitigating anthropogenic climate change.^1^ A broad spectrum of technologies has been developed for molecular CO_2_ capture, including aqueous amine scrubbing systems,^2^ direct air capture (DAC) sorbents,^3,4^ and ionic liquids absorbents.^5,6^ While these approaches have demonstrated practical viability, each exhibits inherent limitations. Aqueous amines often suffer from volatility, oxidative degradation, and significant regeneration energy penalties.^2,7^ Solid sorbents can exhibit high capacity but may require elevated temperatures for regeneration and incur substantial material and operational costs.^3,4^ Ionic liquids and related liquid absorbents offer tunability but frequently display high viscosity, limited mass transfer rates, or sensitivity to moisture.^6^ These trade-offs highlight the need for molecular systems whose reactivity and stability can be rationally optimized at the molecular level.^7^

An ideal molecular CO_2_-capture agent should satisfy multiple criteria simultaneously: (i) rapid kinetic reactivity toward CO_2_ under ambient conditions, (ii) sufficient thermodynamic driving force for reversible or controllable binding, (iii) low regeneration energy, (iv) chemical stability under humid and oxidative environments, and (v) synthetic accessibility and scalability.^3–8^ Achieving this multidimensional balance remains challenging because improvements in one dimension often compromise another.

Carbanions represent a promising class of sorbents for CO_2_ chemisorption^6,8^ and serve as key intermediates in converting CO_2_ into valuable chemicals and fuels.^7,9,10^ Through nucleophilic attack on CO_2_, carbanions form carboxylate adducts via C–C bond formation, enabling chemically selective capture. Ideal carbanions for efficient CO_2_ chemisorption must provide sufficient thermodynamic driving force and favorable kinetics to enable nucleophilic attack under ambient conditions, while maintaining structural stability that allows controlled downstream transformation or reversibility.^5^

Quantum mechanical (QM) modeling has traditionally been used to investigate reaction mechanisms involving CO_2_ and target carbanions, especially when synthesizing such species is experimentally challenging.^11–13^ Data-driven approaches that integrate QM calculations with statistical or machine-learning models offer a robust workflow for exploring limited chemical spaces.^14–16^ However, these forward-screening strategies remain constrained by the need to enumerate candidate structures a priori, and their scalability becomes limited as chemical space grows combinatorially.

Recent advances in generative AI (gen-AI) offer a complementary strategy by enabling inverse molecular design—reversing the traditional mapping from structure → property to property → structure.^17,18^ A variety of generative frameworks, including diffusion models,^19–23^ generative adversarial networks (GAN),^24,25^ and variational autoencoders (VAEs),^26–28^ have been applied to molecular and materials discovery. In VAEs, an encoder transforms molecular representations into a latent distribution, while the decoder reconstructs molecules from sampled latent points. Joint training with property predictors enables property-guided generation within a smooth and manipulable latent space.^18,29–31^

Here, we apply the Hierarchical Variational Encoder (HierVAE)^27^ to generate novel carbanions with enhanced CO_2_ reactivity, evaluated through predicted reaction rate constants (log *k*) at room temperature in dimethyl sulfoxide (DMSO). Reaction rates are inferred from a nucleophilicity prediction model trained via directed message-passing neural networks (D-MPNNs)^32^ on Mayr’s nucleophile database.^33^ By integrating the trained log *k* predictor into the decoder during fine-tuning of a pretrained latent space (from the ChEMBL database^34^), we direct the generative process toward highly reactive carbanions. This workflow allows the successful generation of novel carbanions with high log *k* values, providing a reaction-targeted framework that complements conventional QM-based and forward-screening approaches.

## 2 Methods

### 2.1 CO_2_ Reactivity Based on the Mayr-Patz Equation

The reactivity of nucleophile–electrophile reactions can be described with the Mayr-Patz equation^35^

1 $$\log k=s_{N}\left(E+N\right)$$

where *k* is the rate constant at 20 °C, *N* is the nucleophilicity parameter, *s*_N_ the nucleophile-specific sensitivity parameter, and *E* is the electrophilicity parameter. Ideally, the parameters in eq 1 are derived from experimental measurements. Given the scarcity of experimental carboxylation reaction data, Eckhoff et al. extended the Mayr-Patz equation for nucleophile-CO_2_ reactions using data-driven approach by incorporating QM calculations with supervised learning and uncertainty quantification.^14^ The extended Mayr-Patz equation reads

2 $$\log k_{{\mathrm{CO}}_{2}}=s_{N}s_{E}\left(E+N\right)$$

whereas the electrophilicity parameter *E* = −14.6 and *s*_E_ = 0.81 were calibrated for the Gibbs free energies of 15 carboxylation reactions in DMSO at 20 °C and atmospheric pressure. We note that, unlike the experimentally derived nucleophilicity parameters in Mayr’s database, these electrophilicity parameters for CO_2_ were obtained from data-driven calibration based on QM calculations. Experimental data is only available for one of these reactions, so they have not yet been fully validated against experimental kinetic measurements.^14^ Moreover, because Mayr parameters are solvent-dependent, their transferability beyond DMSO has not yet been established. Consequently, the absolute log *k* values predicted using eq 2 inherit the assumptions and uncertainties associated with this solvent-specific calibration. By applying eq 2, reactions with log *k* > −6 are generally considered observable at room temperature under second-order kinetic conditions in the millimolar concentration regime,^36^ and this threshold should be regarded as a practical rule of thumb rather than a strict physical limit. Conversely, values above log *k* ≈ 8 approach the diffusion-controlled regime, where the reaction rate is controlled by reactant diffusion and the Mayr-Patz equation breaks down.^37^

### 2.2 Prediction of the Nucleophilicity Parameters

Accurate experimental determination of *N* and *s*_N_ remains challenging. Recent machine-learning approaches trained on Mayr’s database have achieved strong performance in predicting nucleophilicity and electrophilicity parameters.^38^ Following this strategy, we trained a D-MPNN model (Chemprop v1.2.0 implementation^32,39^ ) that incorporates both molecular graph features and solvent descriptors to predict Mayr nucleophilicity parameters *N* and *s*_N_. From Mayr’s database, 1039 nucleophiles with valid SMILES were extracted and split into training/validation/test subsets (0.8/0.1/0.1). A 10-fold cross-validation scheme was used, with five ensemble models per fold.

The D-MPNN architecture employed message-passing depth 3 with hidden dimension 300 and mean aggregation. The feed-forward network consisted of two layers with hidden size 300 and ReLU activation; dropout was set to 0.0. Models were trained for a fixed 1000-epoch schedule with batch size 50 using RMSE as the evaluation metric. A learning-rate schedule with init_lr = 1 × 10^–4^, max_lr = 1 × 10^–3^, and final_lr = 1 × 10^–4^ was applied with two warmup epochs. The checkpoint corresponding to the lowest validation root-mean-square error (RMSE) was selected for evaluation. No explicit weight decay regularization was applied; overfitting was mitigated through 10-fold cross-validation and the use of an ensemble of five independently initialized models per fold. Final predictions were obtained by averaging ensemble outputs, and predictive uncertainty was estimated from the standard deviation across ensemble members. Training was performed on NVIDIA GeForce RTX 4090 with CUDA enabled.

### 2.3 Hierarchical Variational Encoder (HierVAE)

HierVAE is a generative framework that constructs molecular graphs using a hierarchical representation of structural motifs. In this approach, frequently occurring substructures extracted from the training set are treated as motifs, which become nodes in a graph representation of the molecule. The decoder generates new molecules by iteratively selecting motifs, predicting where they should attach, and determining the atom-level bonding that finalizes the structure. These steps are carried out through the motif, attachment, and atom layers, allowing the model to build molecules in a chemically coherent manner while efficiently exploring structural diversity.

In this work, we use the HierVAE implementation by Jin et al.,^27^ pretrained on the ChEMBL database to establish a broad and chemically valid latent prior. To guide the model toward CO_2_-reactive carbanions, we incorporate a curated set of active carbanions from Mayr’s database together with an external property predictor that estimates log *k* values from nucleophilicity parameters (Figure 1). During fine-tuning, the model generates batches of new molecules, which are evaluated using activity thresholds and Tanimoto similarity constraints relative to the seed set. Only molecules predicted to be reactive are fed back into training, creating a selective reinforcement loop that gradually shifts the latent probability distribution toward regions associated with high CO_2_ reactivity. Through this integrated process, the model autonomously discovers structurally novel carbanions with enhanced predicted reactivity, effectively coupling the structural expressiveness of HierVAE with the property guidance provided by the nucleophilicity-based scoring function.

**Figure 1:**
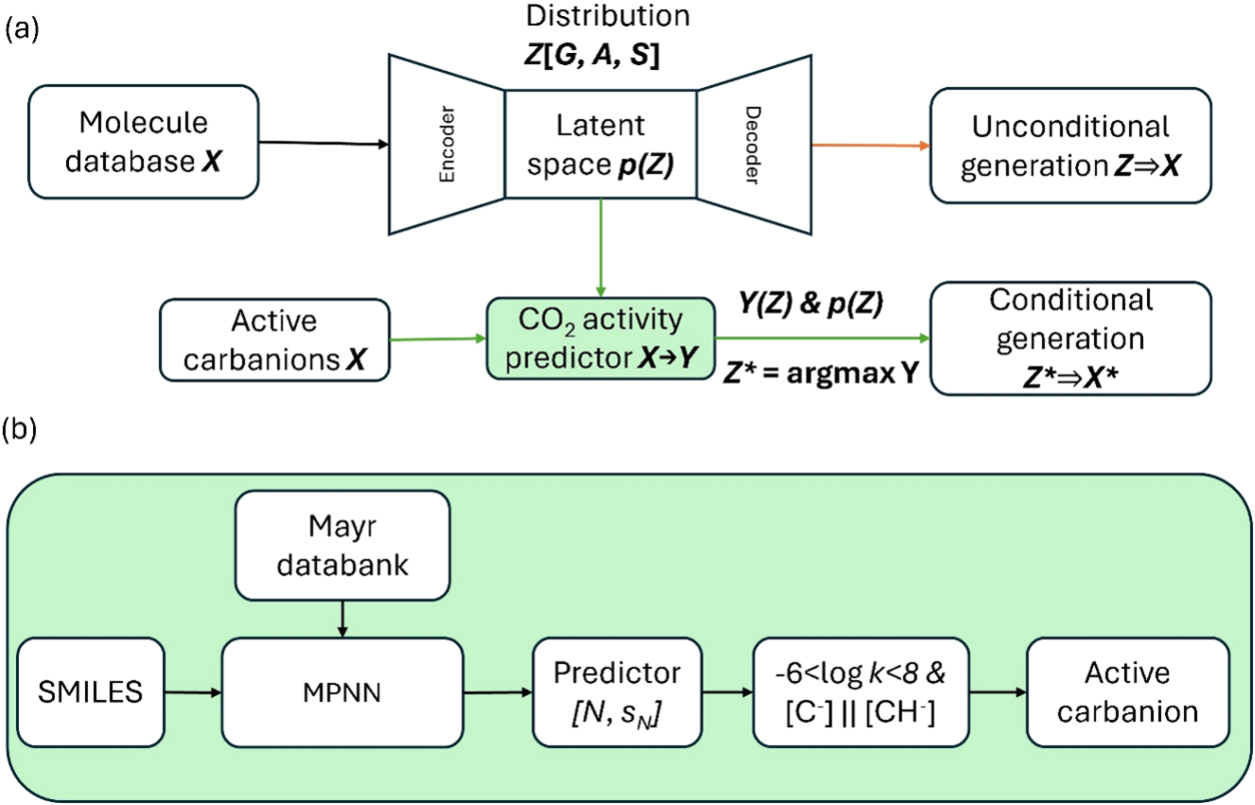
(a) Workflow of the unconditional and conditional generation processes of HierVAE. In latent space *Z*, a molecule structure is represented by a hierarchical graph with motif *S*, attachment *A*, and atom *G* layers. (b) CO_2_ activity predictor for carbanions implemented in this work.

### 2.4 Density Functional Theory Calculations

The density functional theory (DFT) calculation settings are kept consistent with the work of parametrizing *E* and *s*_E_ for CO_2_.^14^ The initial structures of the SMILES strings (carbanions and carboxylates) were first generated using the ETKDG module of RDKit.^40^ Conformers were then optimized using CREST^41^ at GFN2-xTB^42^ level to select the lowest-energy configuration. Geometry optimization and transition state search were performed at B3LYP^43,44^ level of theory with def2-SVPD basis set^45,46^ and D3-type dispersion correction with Beche-Johnson damping^47,48^ using Gaussian 16.^49^ To locate the approximate transition state structure, the relaxed potential energy surface (PES) scan with the step size of 0.05 Å was performed by modifying the C–C distance between carbon atom of CO_2_ and the α carbon atom of the carbanion. The Berny method^50^ was used to find the more accurate transition state structure following the PES scan, and normal-mode analysis was performed to check the presence of a single imaginary frequency. From the TS, the activation energy barrier of the calculated Gibbs free energy in forming carboxylate from CO_2_ and carbanion was computed and converted to log *k*. Goodvibes^51^ was employed to apply the quasi-harmonic correction to the vibrational entropy^52^ computed by Gaussian when calculating the Gibbs free energy. Solvation effect was included by using the SMD solvation model^53^ with parametrization for DMSO. Single-point calculations using def2-TZVPD basis set^45,46,53^ were performed to report the energetic results based on the structures from geometry optimization and transition state search.

## 3 Results and Discussion

### 3.1 Performance of the Nucleophilicity Prediction Model

We first evaluated the performance of the nucleophilicity prediction model trained in this work. The parity plots of the best-performing model (Figure 2) and the averaged metrics over 50 trained models (Table 1) demonstrate that the predictor reliably captures experimental trends in Mayr’s database. For nucleophilicity *N*, the best-performing model achieves a root-mean-square error (RMSE) of 1.848 and a mean-absolute error (MAE) of 1.340 on the test set, comparable to the previously reported MAE of 1.45 by Liu et al.^38^ The sensitivity parameter *s*_N_ is more challenging to predict, largely because 86% of the experimental data fall within the narrow range of 0.5 to 1.0 (Figure S1, SI). Nevertheless, the model obtains an averaged RMSE of 0.114 and an MAE of 0.0759 for *s*_N_, reflecting reasonably strong performance given the compressed distribution of target values. By referring to the underlying data distributions of *N* and *s*_N_, we estimate that 95% of the predicted values fall within ±4.83 for *N* and ±0.223 for *s*_N_ relative to their ground truth values on average. Our best-performing model exceeds these baselines, producing tighter 95% prediction intervals of ±2.96 for *N* and ±0.134 for *s*_N_. This increased accuracy is critical because these predictions directly feed into the computation of the reaction rate constant log *k* during fine-tuning. The model selected for use in the generation pipeline therefore reflects the highest attainable accuracy among the ensemble, ensuring a reliable property signal for steering HierVAE toward more CO_2_-reactive carbanions.

**Figure 2:**
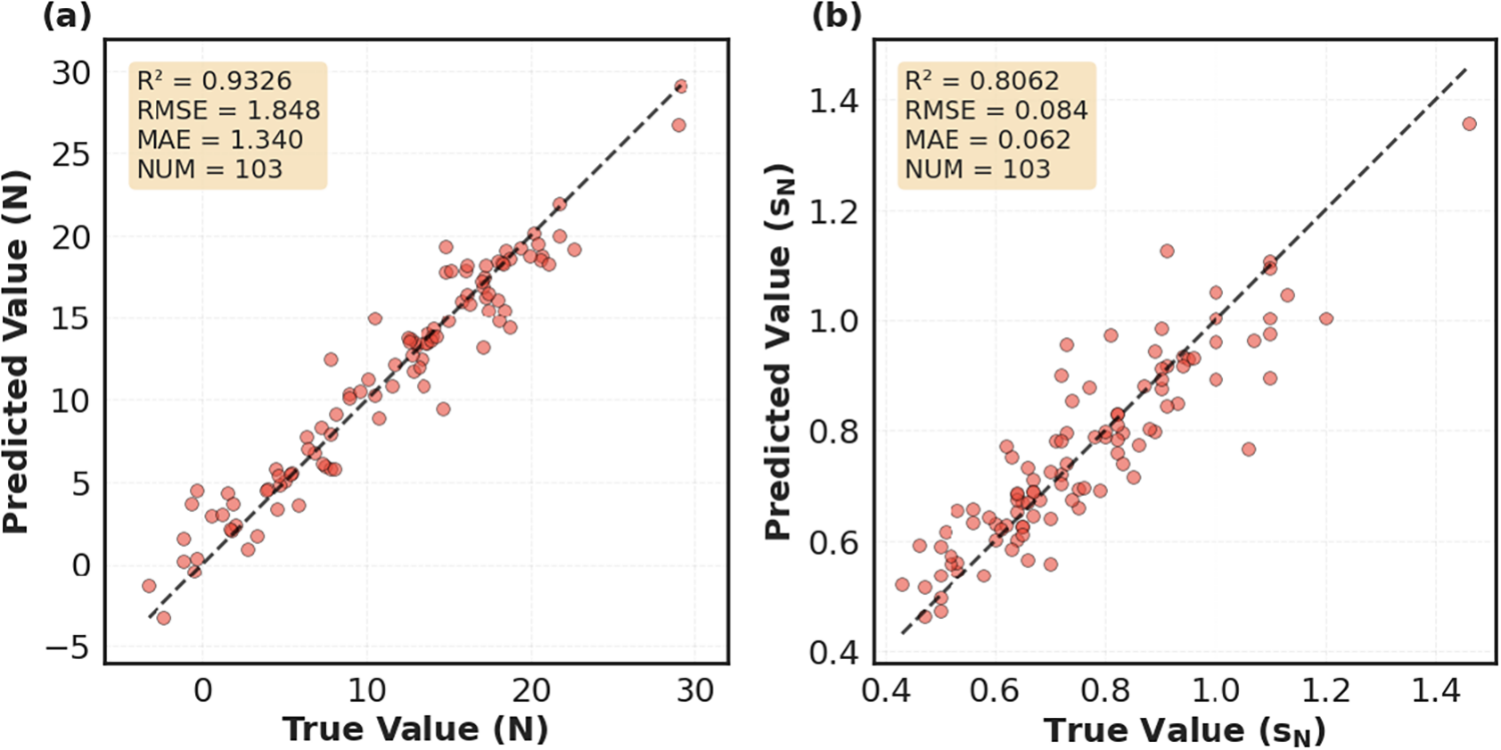
Parity plot of the best performing Chemprop model out of 50 on the test set: (a) the nucleophilicity, *N*; (b) the sensitivity parameter, *s*_N_.

**Table 1:** Average Performance Score of the 50 Trained Chemprop Models of the Prediction of *N* (Range: −3.3 to 29.11) and *s*_*N*_ (Range: 0.43 to 1.46) on the Test Set (103 Molecules)

	metrics	average
*N*	RMSE	2.327 ± 0.282
	MAE	1.615 ± 0.177
*s*_N_	RMSE	0.114 ± 0.017
	MAE	0.0759 ± 0.006

### 3.2 Analysis of the Carbanions Seeds

We next analyzed the structural characteristics of the carbanions used as seeds for fine-tuning, including those taken from Mayr’s database as well as the three reported CO_2_-reactive species MN, MMN, and MSA.^11,13^ Most of the seed carbanions fall into secondary ([CH^–^], 72 structures) and tertiary ([C^–^], 47 structures) categories (Figure S2, SI). Examination of α-substituent combination patterns revealed clear trends in their influence on CO_2_ reactivity, as shown in Figure 3a (plots with individual α-substituent type can be found in Figure S3). When the α-substituent combinations in secondary and tertiary carbanions were mapped against their log *k* distributions, several highly active patterns emerged. In particular, the combinations aryl + ester (Ar + CO2R), aryl + cyano (Ar + CN), aryl + ketone (Ar + COR), and sulfonyl + halide (SO2 + X) yielded the highest average log *k* values, exceeding 6. In contrast, the most common substituent combinations in Mayr’s data set—such as CO2R + CO2R, CO2R + COR, and Ar + SO2R—generally exhibited lower CO_2_ reactivity, falling below the overall average log *k*. Representative examples of these substituent patterns (Figure 3b) further illustrate that substituents with strong electron-withdrawing character can enhance CO_2_ reactivity, whereas overstabilization or excessive delocalization may diminish the nucleophilicity needed for efficient attack on CO_2_. We emphasize that these trends are derived from log *k* values calculated using the CO_2_-specific Mayr–Patz framework and therefore represent model-based structure–reactivity relationships rather than independent mechanistic validation. This seed-set analysis therefore provides important context for evaluating whether the generated structures learned or deviated from the structural motifs associated with high reactivity in experimentally studied carbanions.

**Figure 3:**
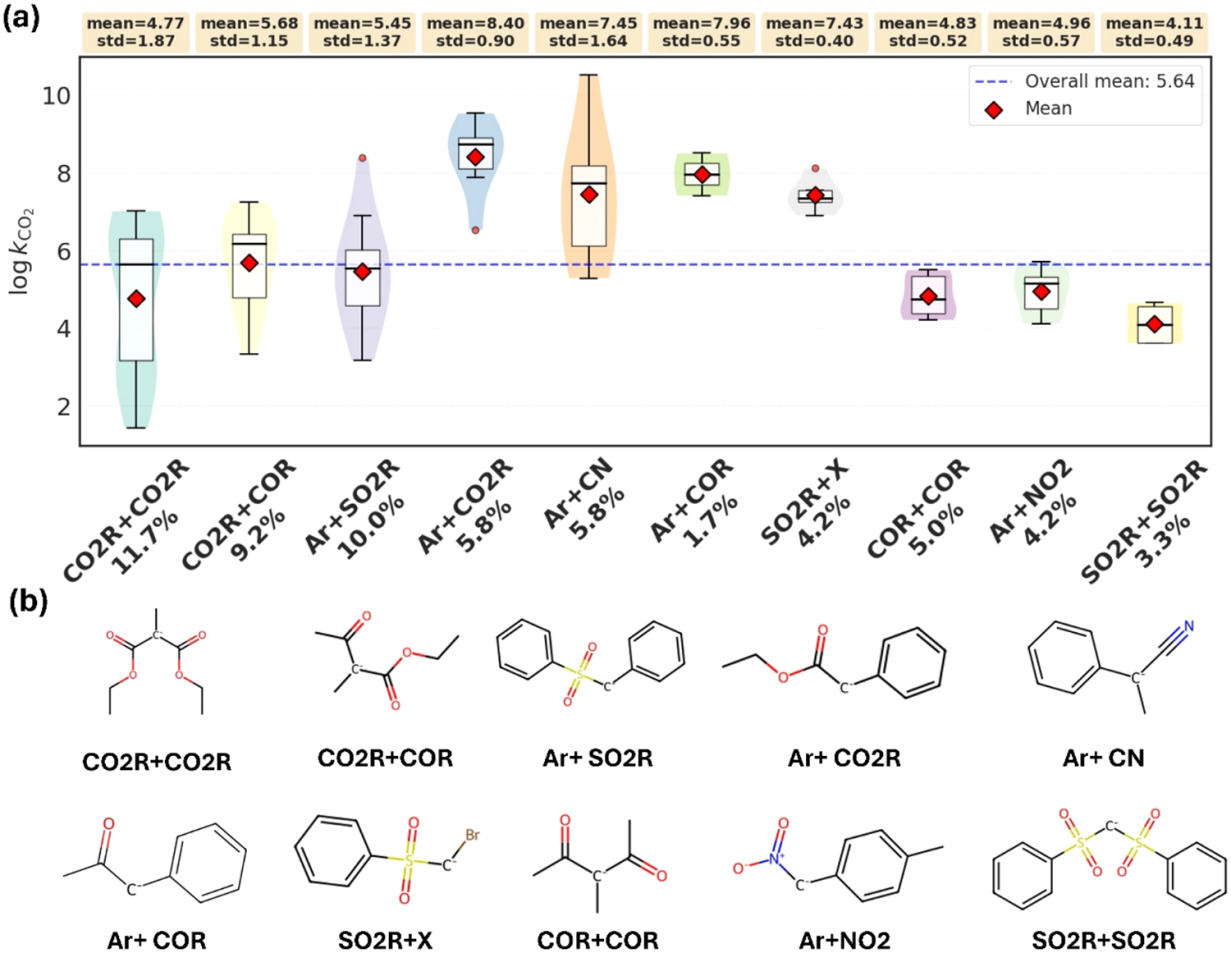
(a) log *k*(CO_2_) distributions of the carbanion seeds (120 in total) with different α-substituent combinations. (b) The representative structures of each combination. Name of the substituent groups: CO2R, ester; COR, ketone; Ar, aryl; CN, cyano; SO2R, sulfonyl; X, halide; NO2: nitro.

### 3.3 Generation of Novel CO_2_ Active Carbanions

During fine-tuning, the HierVAE model rapidly adapted toward producing carbanion structures. The success rate, defined as the ratio of valid carbanions to the total number of sampled molecules, increased dramatically from an initial 0.26 to 98% after only ten epochs (Figure 4). Alongside this improvement, the uniqueness ratio—defined as the fraction of unique carbanions among all generated carbanions—reached a maximum of 42% around epoch five, before gradually declining to around 18% by epoch 15. This trajectory indicates that the model first explores diverse structural regions before eventually converging toward more reactive motifs favored by the scoring function.

**Figure 4:**
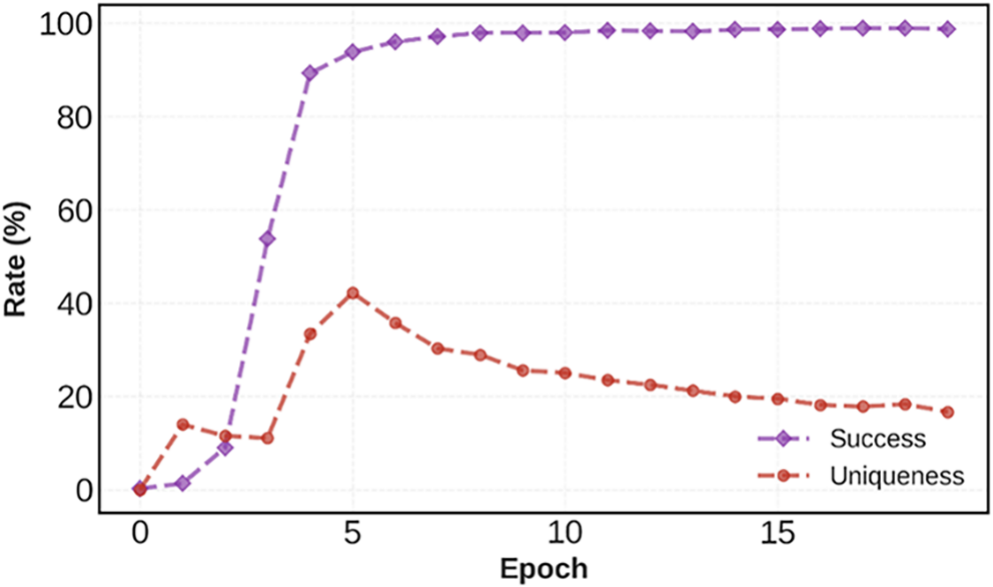
Success and uniqueness rates during the 20 epochs of generation.

A detailed property analysis of the 42,513 generated carbanions shows that the model sampled a wide and chemically meaningful space. The nucleophilicity parameter *N* follows a normal distribution centered at 18.47 (Figure 5a), while the sensitivity parameter *s*_N_ is normally distributed around 0.68, Figure 5b. When combined using the Mayr–Patz equation for CO_2_, the resulting log *k* values span a broad range from approximately −2.34 to 6.82 (Figure 5c). The distribution has an average of 2.01 and a standard deviation of 1.27. The Tanimoto similarity between each generated carbanion and its nearest seed structure follows a distribution centered around 0.31 (Figure 5d). This indicates that the generated molecules are structurally distinct from the seed set and that the model did not simply memorize or reproduce known motifs.

**Figure 5:**
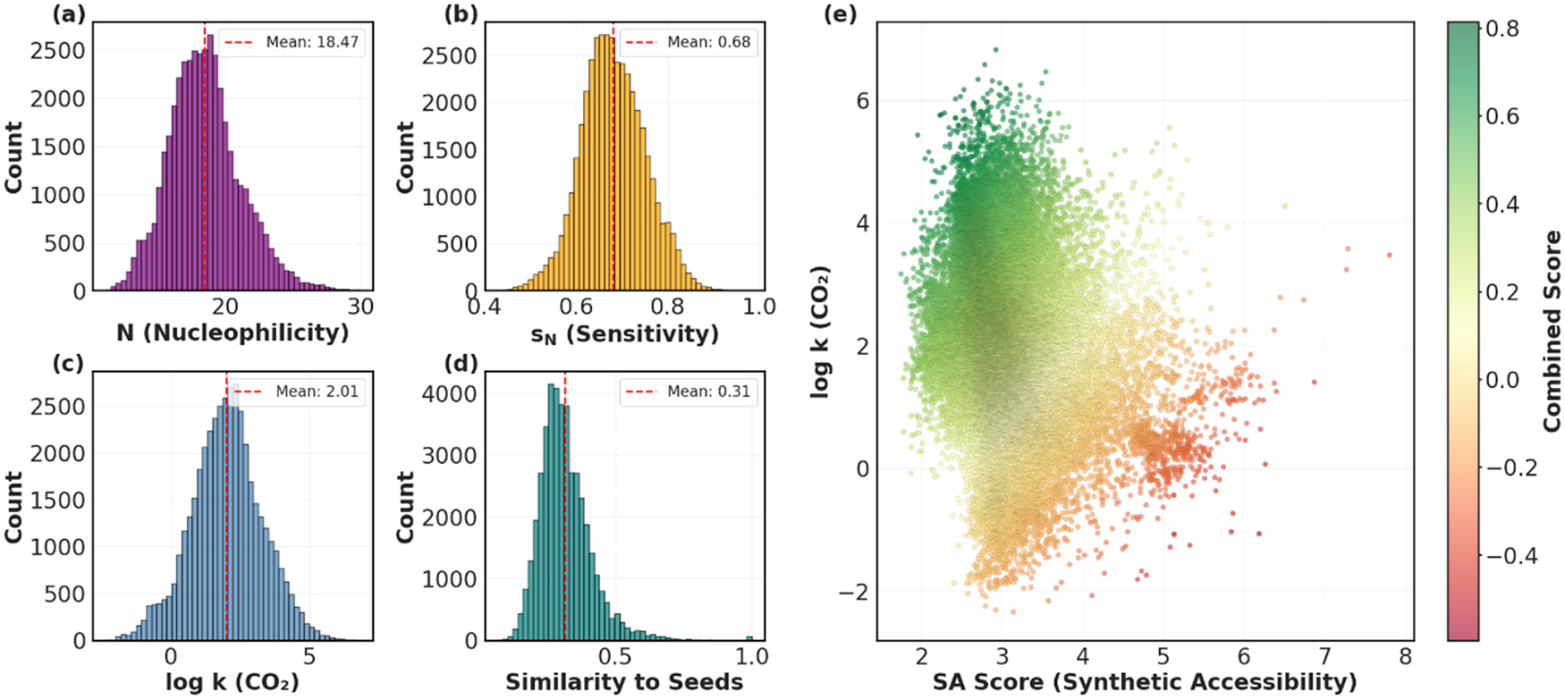
Property distribution of the 42,513 generated novel carbanions: (a) nucleophilicity parameter *N*, (b) sensitivity parameter *s*_N_, (c) log *k* (CO_2_) values, (d) Tanimoto similarity to seed carbanions, and (e) scatter plot of log *k* values and the synthetic accessibility (SA) score, colored by the combined score, defined as normalized­(log *k*)–normalized­(SA).

Because log *k* is derived from predicted *N* and *s*_N_, we further estimated the uncertainty in log *k* induced by model prediction errors. Using standard error propagation for eq 2, and adopting the 95% prediction intervals of ±2.96 for *N* and ±0.134 for *s*_N_ from the best-performing model introduced in Section 3.1, we obtain a propagated uncertainty of σ_log*k*_ ≈ 0.86. This corresponds to a 95% interval of approximately ±1.68 at the mean generated values. For comparison, the generated log *k* distribution spans a range of 9.16 units (−2.34 to 6.82) and has a standard deviation of 1.272 (Figure 5c). Thus, while prediction uncertainty is not negligible relative to the intrinsic spread of log *k*, it remains substantially smaller than the overall dynamic range of the generated set. This indicates that prioritization of highly reactive candidates is statistically robust, whereas marginal differences between closely ranked molecules should be interpreted with appropriate caution.

To assess the practical potential of these molecules, we examined the relationship between the predicted log *k* values and the synthetic accessibility (SA) score, as shown in Figure 5e. The Pearson correlation coefficient between log *k* and SA is −0.215, indicating a weak negative relationship between reactivity and synthetic complexity. By normalizing and combining log *k* and SA into a single metric, two attractive classes of candidates emerge. The first includes molecules with high intrinsic activity (log *k* > 6). The second includes candidates with moderate activity (log *k* > 4) but favorable synthetic accessibility (SA < 3). This combined view highlights the potential to discover not only highly reactive carbanions but also practical ones that balance reactivity with ease of synthesis.

### 3.4 Structural Analysis of the Top 100 Candidates

A detailed structural analysis of the top 100 generated carbanions reveals clear differences between rankings based solely on predicted log *k* and those based on the combined log *k* – SA score. In both ranking schemes, secondary carbanions ([CH^–^]) dominate (Figure 6a). However, ranking strictly by log *k* yields a higher proportion of tertiary carbanions ([C^–^]). This trend likely reflects the increased stabilization associated with more heavily substituted α-centers. When synthetic accessibility is included, however, the ranking shifts toward a greater number of primary carbanions ([CH_2_^–^]), which are generally more feasible to synthesize.

**Figure 6:**
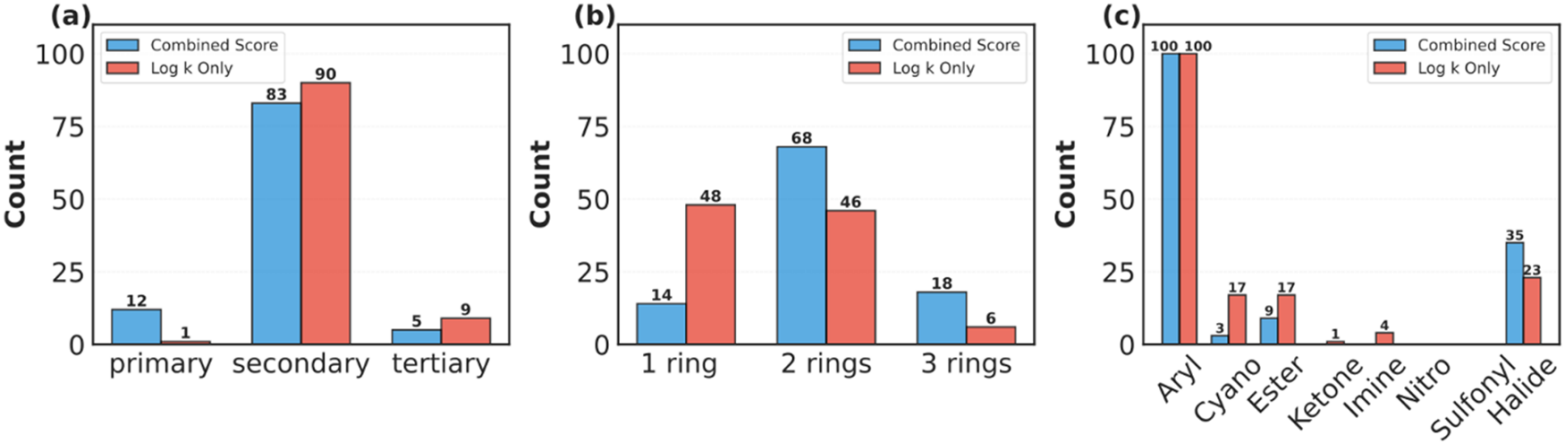
Structural pattern for the top 100 generated novel carbanions ranked by the log *k* value and the combined score with the penalty of synthetic accessibility. (a) Population of primary, secondary and tertiary carbanions, (b) Population of carbanions with one, two and three aromatic rings, and (c) Population of carbanions with different electron withdrawing groups.

Ring-count analysis (Figure 6b) shows that the most reactive carbanions are distributed roughly equally between single-ring and two-ring systems. In contrast, the combined-score ranking includes a higher proportion of molecules containing biphenyl scaffolds. This enrichment is consistent with their low SA scores and commercial availability. Electron-withdrawing groups (EWGs) play a significant role in determining the reactivity of α-carbanions. Across the top candidates, all molecules contain aryl groups (Figure 6c), underscoring their importance in stabilizing the carbanion while maintaining nucleophilicity. The log *k*-based ranking contains a higher frequency of cyano, ester, and imine substituents, which enhance the stabilization of the α-carbon and promote fast nucleophilic attack on CO_2_. These observations align closely with structural patterns observed in the seed set.

Interestingly, nitro and sulfonyl groups—present in the seed carbanions—are largely absent from the top generated candidates. Their overall frequency within the generated data set is low (3.3% for nitro, 1.9% for sulfonyl), and they exhibit below-average reactivity according to the predicted log *k* values (Figure S5), suggesting that their underrepresentation is primarily driven by lower predicted reactivity rather than synthetic accessibility. Halide substituents, although present with moderate frequency, primarily appear on aromatic rings rather than as α-substituents, which differs from their behavior in the seed set. When comparing the top 100 candidates from each ranking scheme, the combined-score list reveals many biphenyl-derived structures with moderate log *k* values (4.6–6.8) and low SA scores (<2.5), as shown in Figure 7a-c. In contrast, the log *k*-focused ranking contains more structurally diverse carbanions with cyano, ester, or ketone group, but at the cost of higher synthetic complexity (SA > 3.5), as shown in Figure 7b–d. The α-substituent combination of aryl with these groups also tend to yield higher log *k* values (Figure S6). Together, these results demonstrate that property-guided generative modeling can produce chemically diverse and synthetically relevant carbanions while capturing the structural trends needed for strong CO_2_ reactivity. We emphasize that these substituent–reactivity relationships are inferred from the model-predicted log *k* values and reflect the structure–reactivity patterns learned from Mayr’s database; accordingly, they should be interpreted within the domain of applicability of the trained predictor rather than as independent mechanistic validation.

**Figure 7:**
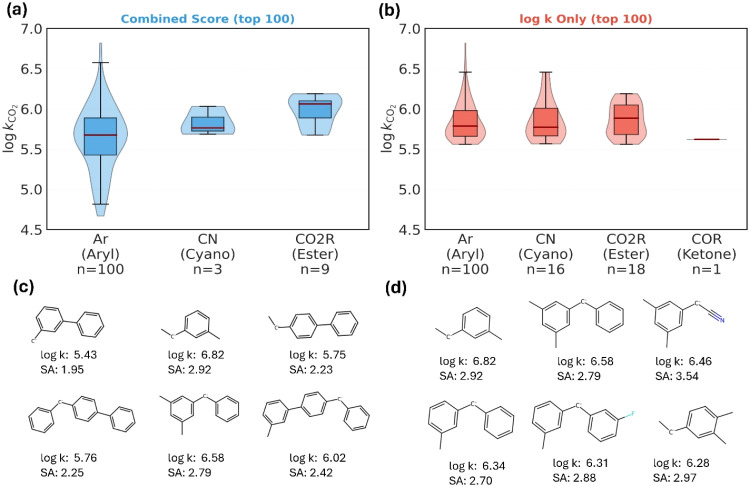
Top panel: structural pattern analysis of the top 100 candidates ranked by (a) log *k* only and (b) the combined score with the SA penalty. Bottom panel: representative structures for (c) log *k* only ranking and (d) combined score ranking.

### 3.5 DFT Validation

To evaluate the reliability of the ML-predicted log *k* values, DFT calculations were performed for the top 100 candidates ranked by predicted reactivity. Among these, 20 carbanions—primarily belonging to Ar + alkyl (18) and Ar + Ar (2) substitution patterns—exhibited effectively barrierless C–C bond formation during the carboxylation reaction at the level of theory of B3LYP/def2-TZVPD with D3 correction, as indicated by flat PES scans without a well-defined transition state. For the remaining 80 carbanions where transition states could be successfully identified, activation barriers were computed and converted to log *k* values for comparison with ML predictions.

Across the full set of 80 carbanions, the correlation between ML-predicted and DFT-derived log *k* values is weak (Pearson *r* = −0.19), indicating that the predictive performance is not uniform across substitution patterns. The RMSE of 1.596 is comparable to error reported for the data set used to calibrate the CO_2_-specific *E* and *s*_N_ parameters.^14^ The largest deviations occur for Ar + alkyl and Ar + Ar systems (Table 2), which also correspond to the combinations in the 20 barrierless carbanions. A plausible explanation is that the electrophilicity parameters of CO_2_ were calibrated primarily using carbanions stabilized by strong EWGs,^14^ potentially limiting transferability to weakly stabilized systems such as monoaryl alkyl or diaryl carbanions. In contrast, carbanions bearing strong EWGs (e.g., CN and CO2R) show significantly improved agreement between ML predictions and DFT calculations. For the subset with |log *k*|_diff_ < 0.5 which comprises Ar + CN (11) and Ar + CO2R (4), the parity plot yields a Pearson correlation coefficient of 0.675 with MAE = 0.19 and RMSE = 0.23 (Figure 8a,b). These results indicate that the Chemprop-based predictor performs most reliably for electronically stabilized carbanions consistent with the underlying parametrization domain.

**Figure 8:**
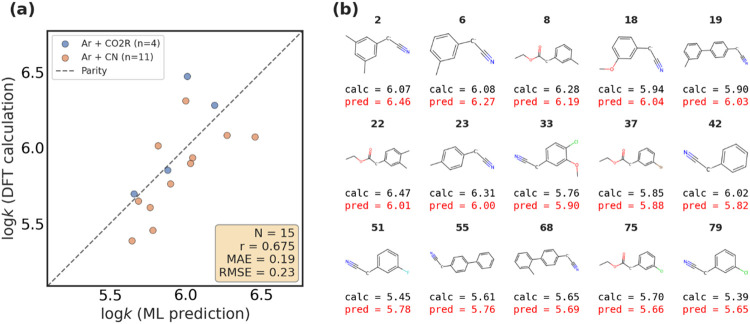
(a) Parity plot and (b) structures of the subset with |log *k*|_diff_ < 0.5 between the DFT calculated log *k* and the ML prediction.

**Table 2:** RMSE and MAE of log *k* Difference between ML Prediction and DFT Calculations for Different Categories in the 80 Carbanions with Identified TS Structure for Carboxylation Reactions

category	*n*	RMSE	MAE
Ar + CN	16	1.164	0.668
Ar + COR	1	1.193	1.193
Ar + CO2R	18	1.615	1.297
Ar + alkyl	4	1.614	1.577
Ar + Ar	41	1.733	1.621
**All**	80	1.596	1.350

Taken together, the DFT validation reveals a clear domain-of-applicability effect: predictive accuracy is highest for strongly stabilized nucleophiles resembling those used in the CO_2_ parameter calibration for the extended Mayr-Patz equation, while deviations increase for weakly stabilized or sterically distinct systems.

### 3.6 Limitation and Scope of Applicability

The present framework prioritizes candidate carbanions based on predicted kinetic reactivity (log *k*) derived from the Mayr–Patz relationship. While log *k* reflects the activation barrier for nucleophilic attack, overall CO_2_ reaction feasibility may also depend on thermodynamic factors such as adduct stability.^16^ High predicted log *k* values therefore indicate favorable kinetics but do not necessarily guarantee favorable equilibrium binding or practical capture performance.

Moreover, the CO_2_-specific Mayr–Patz parametrization employed in this work was calibrated in DMSO at 20 °C using Gibbs free energies from a limited set of carboxylation reactions obtained from QM calculations, primarily involving electronically stabilized nucleophiles. Although the formal structure of the Mayr–Patz equation is general, the quantitative parameters (*E* and *s*_E_) adopted here therefore define a solvent-specific and electronically constrained calibration domain derived from QM-based data. When applied to extrapolative design of new carbanions, the model should accordingly be interpreted within this solvent, temperature, and nucleophile-class regime, and its transferability beyond these conditions is not guaranteed.

In addition, the Mayr–Patz model does not explicitly encode steric accessibility or three-dimensional geometric constraints. Although log *k* captures electronic contributions to the transition state, steric congestion may influence formation of the reactive encounter complex or the stability of the resulting adduct. As indicated by the DFT validation, predictive reliability varies across substitution patterns: agreement is strongest for electronically stabilized systems and weaker for structurally distinct or weakly stabilized carbanions. These results define the current domain of applicability of the model. Future extensions integrating thermodynamic descriptors and explicit structural metrics could provide a more comprehensive assessment of CO_2_–carbanion reactivity.

## 4 Conclusions

This work demonstrates that property-guided generative AI can successfully design novel carbanions exhibiting strong CO_2_ reactivity and reasonable synthetic accessibility. The nucleophilicity predictor developed using a D-MPNN model achieves high accuracy (*R* ^2^ = 0.93 for *N*, 0.81 for *s*_N_), enabling reliable computation of reaction rate constants during HierVAE fine-tuning. The generative process ultimately yields a 98% success rate and 18% molecular uniqueness across 20 epochs. Structural analysis reveals that highly reactive carbanions typically feature secondary carbon centers with aryl α-substituents and strong electron-withdrawing groups such as cyano and ester moieties—consistent with experimentally established design principles. Introducing synthetic accessibility constraints shifts the focus toward biphenyl-derived carbanions, which maintain moderate activity while offering practical synthetic advantages.

DFT validation of the highest-ranked reactive candidates reveals a clear domain-of-applicability effect. Agreement between ML-predicted and DFT-derived log *k* values is strongest for electronically stabilized carbanions bearing strong electron-withdrawing groups (e.g., cyano and ester substituents), whereas larger deviations occur for weakly stabilized or sterically distinct systems. These results indicate that predicted log *k* values should be interpreted as relative indicators of nucleophilic attack propensity within the calibrated electronic domain of the CO_2_-specific Mayr–Patz parametrization, rather than as universally quantitative rate predictions.

Overall, this study highlights the potential of coupling generative AI with physically motivated reactivity models to enable reaction-targeted inverse design. Future integration of thermodynamic descriptors, solvent-transferability corrections, and explicit steric metrics will further strengthen the predictive scope of this framework for CO_2_ chemisorption chemistry.

## Supplementary Material



## Data Availability

Data and code are available openly at https://github.com/bo-li/prop_guided_co2_carbanion, including the training data for chemprop, seed carbanions, the whole data set of the generated carbanions, the code and scripts for the training, finetuning and analyzing the results, and the geometry coordinates and input examples of the DFT calculations. A demonstration Jupyter notebook is included for the whole workflow.

## Notes

Distribution of nucleophilicity and sensitivity parameters for the 1039 nucleophiles extracted from Mary’s database. Strutural pattern and log *k* distribution of the seed carbanions; distribution of SA score, combined score, and log *k* for the full 42,513 generated novel carbanions; structures of the top 100 candidates with log *k* and combined score ranking; DFT calculated log *k* values of the 80 carbanions (PDF)

## References

[1] Vitillo J. G., Smit B., Gagliardi L. (2017). Introduction: Carbon
Capture and Separation. Chem. Rev..

[2] Meng F., Meng Y., Ju T., Han S., Lin L., Jiang J. (2022). Research Progress of Aqueous Amine
Solution for CO_2_ Capture:
A Review. Renewable Sustainable Energy Rev..

[3] Qiu L., Mokhtarinori N., Liu H., Jiang D.-e., Yang Z., Dai S. (2025). The Carbon Challenge:
Design, Synthesis, and Chemisorption Behavior
of Solid Sorbents in Direct Air Capture of Carbon Dioxide. Mater. Today Energy.

[4] Siegelman R. L., Kim E. J., Long J. R. (2021). Porous Materials
for Carbon Dioxide
Separations. Nat. Mater..

[5] Zhao Z., Wang K., Tao H., Zhang Z., Lin W., Xiao Q., Jiang L., Li H., Wang C. (2025). Thermodynamic
Regulation of Carbon Dioxide Capture by Functionalized Ionic Liquids. Chem. Soc. Rev..

[6] Qiu L., Li E., Ke T., Wang Q., Tong Y., Li B., Liu H., Jiang D.-e., Mahurin S. M., Yang Z., Dai S. (2025). Frontiers
of Ionic Liquids in Carbon Dioxide Separation and Valorization. Chem. Rev..

[7] Siegel R. E., Pattanayak S., Berben L. A. (2023). Reactive Capture of CO_2_: Opportunities and
Challenges. ACS Catal..

[8] Fu Y., Yang Z., Mahurin S. M., Dai S., Jiang D.-e. (2022). Ionic Liquids
for Carbon Capture. MRS Bull..

[9] Liu X.-F., Zhang K., Tao L., Lu X.-B., Zhang W.-Z. (2022). Recent
Advances in Electrochemical Carboxylation Reactions using Carbon Dioxide. Green Chem. Eng..

[10] Artz J., Müller T. E., Thenert K., Kleinekorte J., Meys R., Sternberg A., Bardow A., Leitner W. (2018). Sustainable
Conversion of Carbon Dioxide: An Integrated Review of Catalysis and
Life Cycle Assessment. Chem. Rev..

[11] Qiu L., Li B., Hu J., Ganesan A., Pramanik S., Damron J. T., Li E., Jiang D.-e., Mahurin S. M., Popovs I. (2024). Cascade
CO_2_ Insertion in Carbanion Ionic Liquids Driven by Structure
Rearrangement. J. Am. Chem. Soc..

[12] Li B., Fu Y., Yang Z., Dai S., Jiang D.-e. (2024). Intermolecular Proton
Transfer Enabled Reactive CO_2_ Capture by the Malononitrile
Anion. J. Phys. Chem. B.

[13] Suo X., Fu Y., Do-Thanh C.-L., Qiu L.-Q., Jiang D.-e., Mahurin S. M., Yang Z., Dai S. (2022). CO_2_ Chemisorption Behavior
in Conjugated Carbanion-Derived Ionic Liquids via Carboxylic Acid
Formation. J. Am. Chem. Soc..

[14] Eckhoff M., Bublitz K. L., Proppe J. (2025). Unveiling
CO_2_ Reactivity
with Data-Driven Methods. Digit. Discovery.

[15] Franceschi P., Nicoletti C., Bonetto R., Bonchio M., Natali M., Dell’Amico L., Sartorel A. (2021). Basicity as a Thermodynamic Descriptor
of Carbanions Reactivity with Carbon Dioxide: Application to the Carboxylation
of α,β-Unsaturated Ketones. Front.
Chem..

[16] Eckhoff M., Deolka S., Garcia-Roca A., Meynberg L., Seidel L., Sigman M. S., Proppe J. (2025). Predicting
the Stability of Base-Mediated
C–H Carboxylation Adducts Using Data Science Tools. Angew. Chem., Int. Ed..

[17] van
den Broek R. L., Patel S., van Westen G. J. P., Jespers W., Sherman W. (2025). In Search of Beautiful Molecules:
A Perspective on Generative Modeling for Drug Design. J. Chem. Inf. Model..

[18] Özçelik R., Brinkmann H., Criscuolo E., Grisoni F. (2025). Generative Deep Learning
for de Novo Drug Design—A Chemical Space Odyssey. J. Chem. Inf. Model..

[19] Weiss T., Yanes E. M., Chakraborty S., Cosmo L., Bronstein A. M., Gershoni-Poranne R. (2023). Guided Diffusion
for Inverse Molecular Design. Nat. Comput. Sci..

[20] Le T., Cremer J., Clevert D.-A., Schütt K. T. (2025). Equivariant
Diffusion for Structure-Based De Novo Ligand Generation with Latent-Conditioning. J. Cheminf..

[21] Huang L., Xu T., Yu Y., Zhao P., Chen X., Han J., Xie Z., Li H., Zhong W., Wong K.-C., Zhang H. (2024). A Dual Diffusion
Model Enables 3D Molecule Generation and Lead Optimization Based on
Target Pockets. Nat. Commun..

[22] Duan C., Du Y., Jia H., Kulik H. J. (2023). Accurate
Transition State Generation
with an Object-Aware Equivariant Elementary Reaction Diffusion Model. Nat. Comput. Sci..

[23] Xu, M. ; Yu, L. ; Song, Y. ; Shi, C. ; Ermon, S. ; Tang, J. Geodiff: A Geometric Diffusion Model for Molecular Conformation Generation. 2022, arXiv:2203.02923. arXiv.org e-Printarchive. https://arxiv.org/abs/2203.02923.

[24] Therrien F., Sargent E. H., Voznyy O. (2025). Using GNN
Property Predictors as
Molecule Generators. Nat. Commun..

[25] Prykhodko O., Johansson S. V., Kotsias P.-C., Arús-Pous J., Bjerrum E. J., Engkvist O., Chen H. (2019). A De Novo Molecular
Generation Method using Latent Vector Based Generative Adversarial
Network. J. Cheminf..

[26] Jin, W. ; Barzilay, R. ; Jaakkola, T. Junction Tree Variational Autoencoder for Molecular Graph Generation. In Proceedings of the 35th International Conference on Machine Learning, Drug Discovery; PMLR, 2018; pp 2323–2332.

[27] Jin, W. ; Barzilay, R. ; Jaakkola, T. In Hierarchical Generation of Molecular Graphs using Structural Motifs; Proceedings of the 37th International Conference on Machine Learning PMLR, 2020; pp 4839–4848.

[28] Gómez-Bombarelli R., Wei J. N., Duvenaud D., Hernández-Lobato J. M., Sánchez-Lengeling B., Sheberla D., Aguilera-Iparraguirre J., Hirzel T. D., Adams R. P., Aspuru-Guzik A. (2018). Automatic
Chemical Design Using a Data-Driven Continuous Representation of Molecules. ACS Cent. Sci..

[29] Shino Y., Kaneko H. (2025). Improving Molecular Design with Direct
Inverse Analysis
of QSAR/QSPR Model. Mol. Inform..

[30] Strandgaard M., Linjordet T., Kneiding H., Burnage A. L., Nova A., Jensen J. H., Balcells D. (2025). A Deep Generative Model
for the Inverse
Design of Transition Metal Ligands and Complexes. JACS Au.

[31] Datta J., Nadimpally A., Koratkar N., Datta D. (2025). Generative AI for Discovering
Porous Oxide Materials for Next-Generation Energy Storage. Cell Rep. Phys. Sci..

[32] Yang K., Swanson K., Jin W., Coley C., Eiden P., Gao H., Guzman-Perez A., Hopper T., Kelley B., Mathea M. (2019). Analyzing
Learned Molecular Representations for Property Prediction. J. Chem. Inf. Model..

[33] Mayr, H. O. ; Armin, R. Mayr’s Reactivity Database. https://www.cup.lmu.de/oc/mayr/reaktionsdatenbank2/. (accessed January 11, 2025).

[34] Gaulton A., Bellis L. J., Bento A. P., Chambers J., Davies M., Hersey A., Light Y., McGlinchey S., Michalovich D., Al-Lazikani B., Overington J. P. (2012). ChEMBL:
A Large-Scale Bioactivity Database for Drug Discovery. Nucleic Acids Res..

[35] Mayr H., Patz M. (1994). Scales of Nucleophilicity and Electrophilicity: A System for Ordering
Polar Organic and Organometallic Reactions. Angew. Chem., Int. Ed..

[36] Mayr H. (2015). Reactivity
Scales for Quantifying Polar Organic Reactivity: the Benzhydrylium
Methodology. Tetrahedron.

[37] Ammer J., Nolte C., Mayr H. (2012). Free Energy
Relationships for Reactions
of Substituted Benzhydrylium Ions: From Enthalpy over Entropy to Diffusion
Control. J. Am. Chem. Soc..

[38] Liu Y., Yang Q., Cheng J., Zhang L., Luo S., Cheng J.-P. (2023). Prediction of Nucleophilicity
and Electrophilicity
Based on a Machine-Learning Approach. ChemPhysChem.

[39] Heid E., Greenman K. P., Chung Y., Li S.-C., Graff D. E., Vermeire F. H., Wu H., Green W. H., McGill C. J. (2024). Chemprop:
A Machine Learning Package for Chemical Property Prediction. J. Chem. Inf. Model..

[40] Landrum, G. ; Tosco, P. ; Kelley, B. ; Rodriguez, R. ; Cosgrove, D. ; Vianello, R. ; sriniker; Gedeck, P. ; Jones, G. ; Kawashima, E. RDKit: Open-source cheminformatics. https://www.rdkit.org.

[41] Pracht P., Grimme S., Bannwarth C., Bohle F., Ehlert S., Feldmann G., Gorges J., Müller M., Neudecker T., Plett C. (2024). CREST—A
Program
for the Exploration of Low-energy Molecular Chemical Space. J. Chem. Phys..

[42] Bannwarth C., Ehlert S., Grimme S. (2019). GFN2-xTB—an
Accurate and Broadly
Parametrized Self-Consistent Tight-Binding Quantum Chemical Method
with Multipole Electrostatics and Density-Dependent Dispersion Contributions. J. Chem. Theory Comput..

[43] Becke A. D. (1988). Density-Functional
Exchange-Energy Approximation with Correct Asymptotic Behavior. Phys. Rev. A.

[44] Lee C. T., Yang W. T., Parr R. G. (1988). Development of the
Colle-Salvetti
Correlation-Energy Formula into a Functional of the Electron-Density. Phys. Rev. B.

[45] Weigend F., Ahlrichs R. (2005). Balanced Basis Sets of Split Valence, Triple Zeta Valence
and Quadruple Zeta Valence Quality for H to Rn: Design and Assessment
of Accuracy. Phys. Chem. Chem. Phys..

[46] Rappoport D., Furche F. (2010). Property-Optimized
Gaussian Basis Sets for Molecular
Response Calculations. J. Chem. Phys..

[47] Grimme S., Ehrlich S., Goerigk L. (2011). Effect of the Damping Function in
Dispersion Corrected Density Functional Theory. J. Comput. Chem..

[48] Grimme S., Antony J., Ehrlich S., Krieg H. (2010). A Consistent and Accurate *ab initio* Parametrization of Density Functional Dispersion
Correction (DFT-D) for the 94 Elements H-Pu. J. Chem. Phys..

[49] Frisch, M. J. ; Trucks, G. W. ; Schlegel, H. B. ; Scuseria, G. E. ; Robb, M. A. ; Cheeseman, J. R. ; Scalmani, G. ; Barone, V. ; Petersson, G. A. ; Nakatsuji, H. Gaussian 16 Rev. C.01 Wallingford, CT, 2016.

[50] Schlegel H.
B. (1982). Optimization
of Equilibrium Geometries and Transition Structures. J. Comput. Chem..

[51] Luchini G., Alegre-Requena J., Funes-Ardoiz I., Paton R. (2020). GoodVibes: Automated
Thermochemistry for Heterogeneous Computational Chemistry Data. F1000Research.

[52] Grimme S. (2012). Supramolecular
Binding Thermodynamics by Dispersion-Corrected Density Functional
Theory. Chem. - Eur. J..

[53] Marenich A. V., Cramer C. J., Truhlar D. G. (2009). Universal Solvation Model Based on
Solute Electron Density and on a Continuum Model of the Solvent Defined
by the Bulk Dielectric Constant and Atomic Surface Tensions. J. Phys. Chem. B.

