# Supplementary material for: Discovering CO2–Reactive Carbanions via Property-Guided Generative AI
Source: J Chem Inf Model. 2026 Jun 10;66(12):6983–91. doi: 10.1021/acs.jcim.6c00680 (PMC13292209; doi:10.1021/acs.jcim.6c00680)
Supplement: Supplementary file 1 [file ci6c00680_si_001.pdf]

Discovering CO_2-Reactive Carbanions via Property-Guided Generative AI

Bo Li[a]* and De-en Jiang[a]*

[a] Department of Chemical and Biomolecular Engineering, Vanderbilt University, Nashville, TN 37235, USA

* Corresponding authors: bo.li.2@vanderbilt.edu; de-en.jiang@vanderbilt.edu

Figure S1. Distribution of the (a) nucleophilicity N and (b) sensitivity s_N for the 1039 nucleophiles extracted from Mary's database......2

Figure S2. Distribution of the structural pattern for the 120 seeds carbanions: (a) frequency of each α-substituent type, and (b) frequency of α-substituent degree......3

Figure S3. Distribution of log *k* for the 120 seeds carbanions with the α-substituent types. 3

Figure S4. Distribution of (a) SA score and (b) the combined score for the 42,513 generated carbanions. 4

Figure S5. Distribution of log *k* for the 42,513 generated novel carbanions with α-substituent types. 5

Figure S6. Distribution of log *k* for the 42,513 generated novel carbanions with α-substituent combinations. 5

Figure S7. Structures for the top 100 generated novel carbanions ranked by log *k*. 6

Figure S8. Structures for the top 100 generated novel carbanions ranked by the combined score of log *k* penalized by synthetic accessibility. 7

Table S1. DFT calculated and ML predicted log *k* values of the 80 carbanions. 8

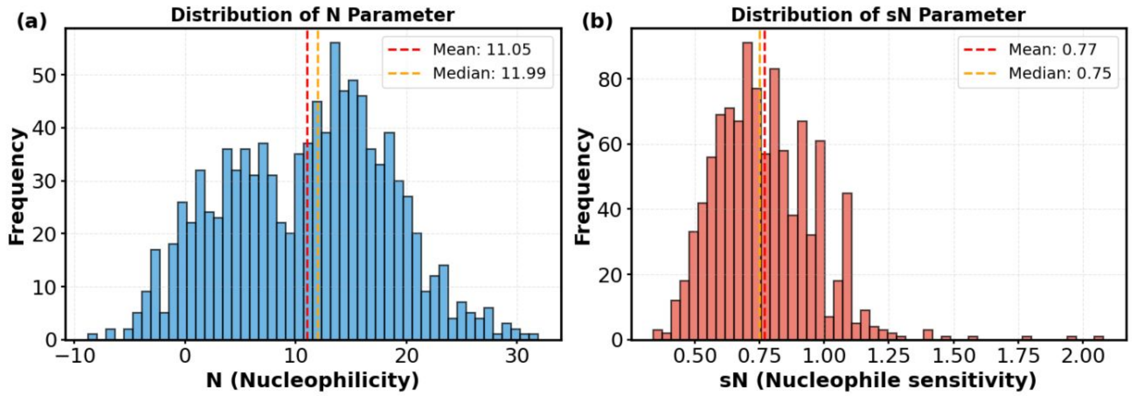

Figure S1. Distribution of the (a) nucleophilicity N and (b) sensitivity s_N for the 1039 nucleophiles extracted from Mary's database.

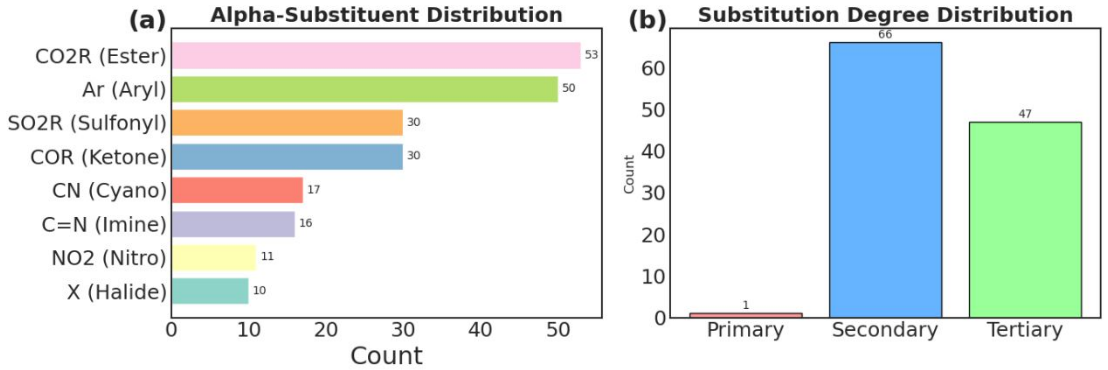

Figure S2. Distribution of the structural pattern for the 120 seeds carbanions: (a) frequency of each α-substituent type, and (b) frequency of α-substituent degree.

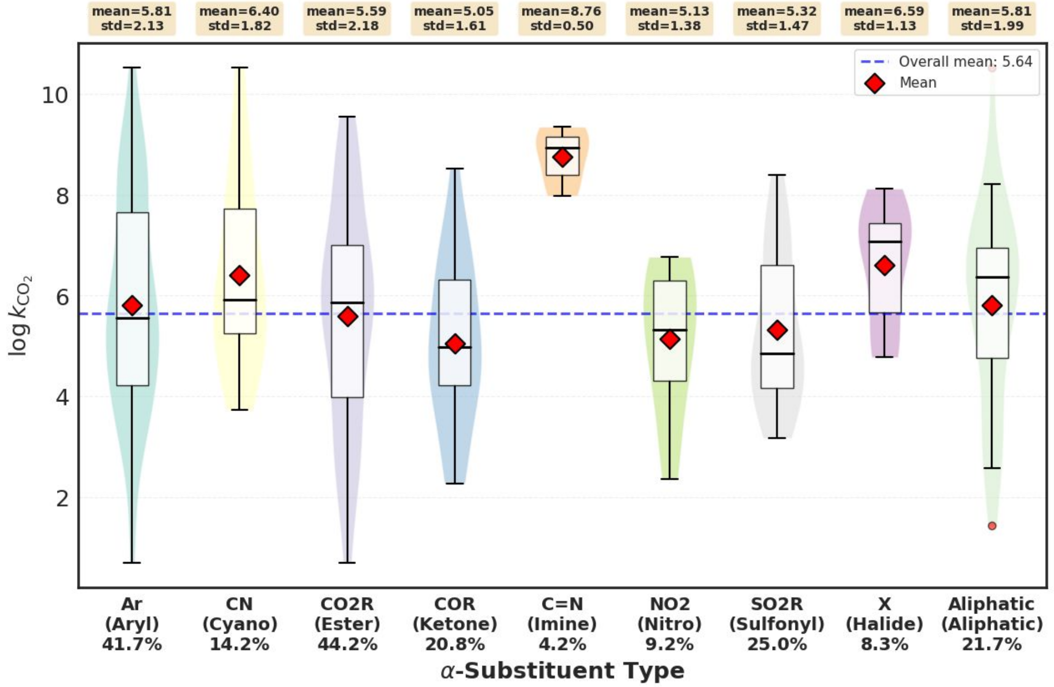

Figure S3. Distribution of log *k* for the 120 seeds carbanions with the α-substituent types.

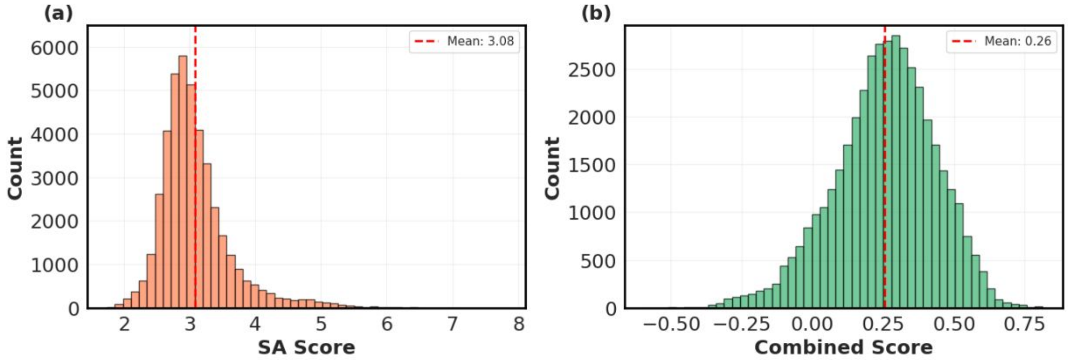

Figure S4. Distribution of (a) SA score and (b) the combined score for the 42,513 generated carbanions.

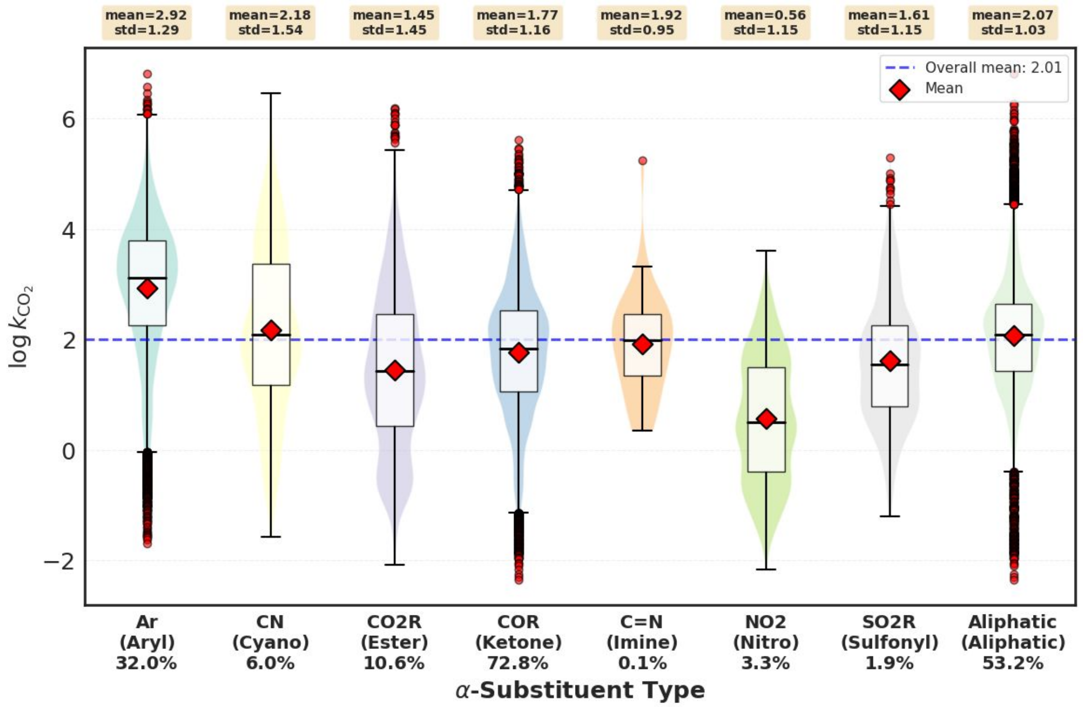

Figure S5. Distribution of log *k* for the 42,513 generated novel carbanions with α-substituent types.

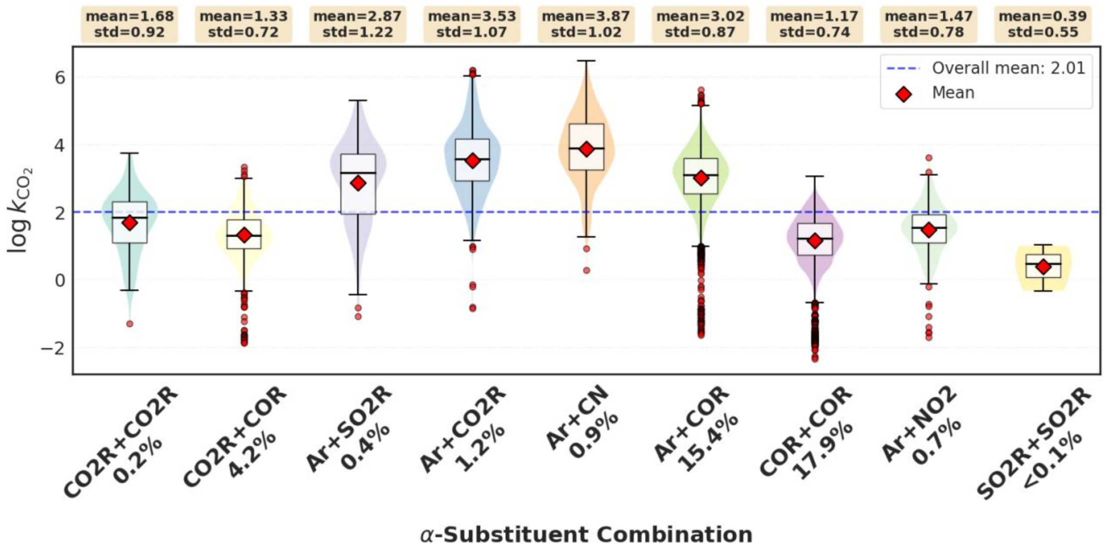

Figure S6. Distribution of log *k* for the 42,513 generated novel carbanions with α-substituent combinations.

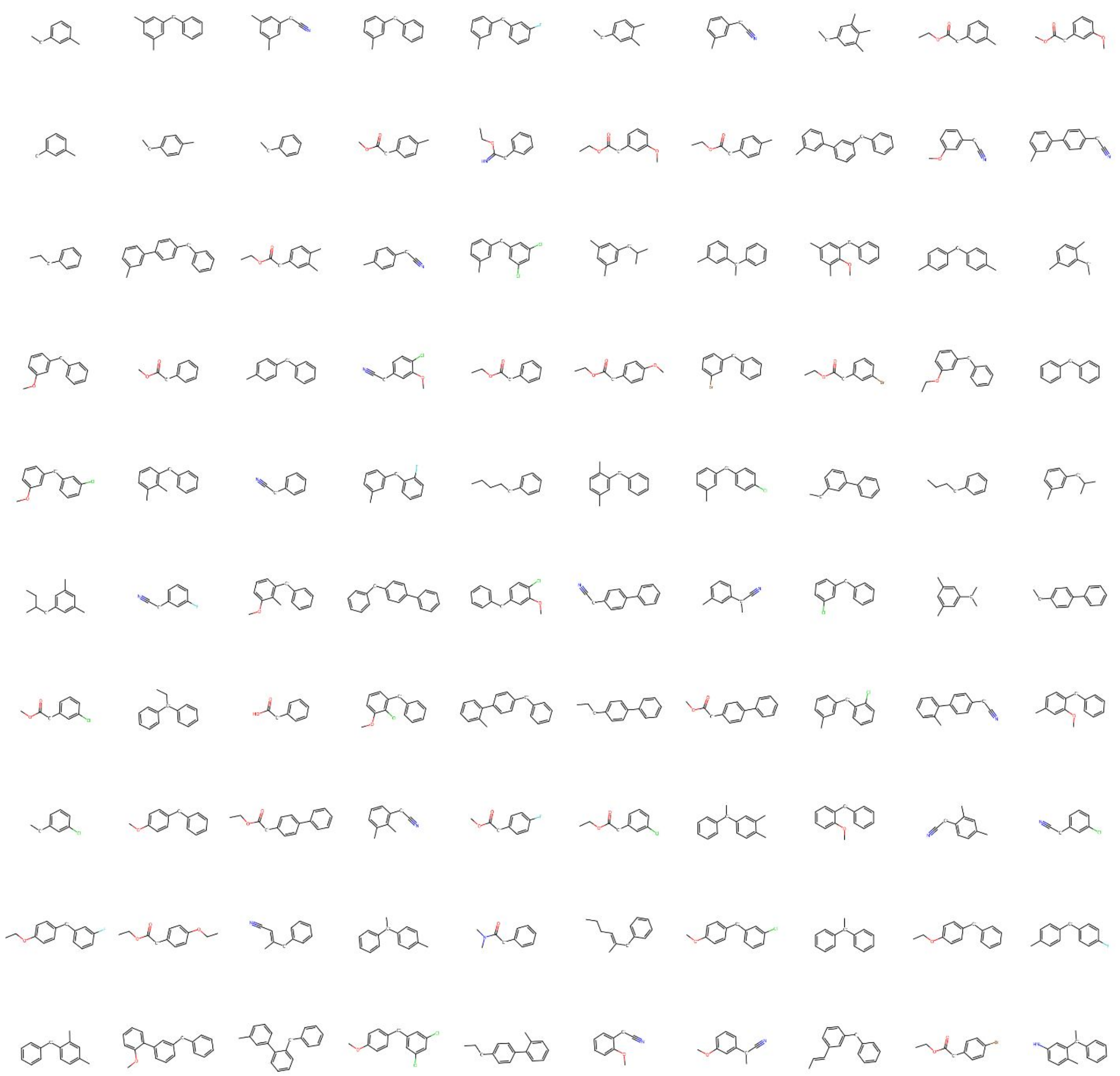

Figure S7. Structures for the top 100 generated novel carbanions ranked by log *k*.

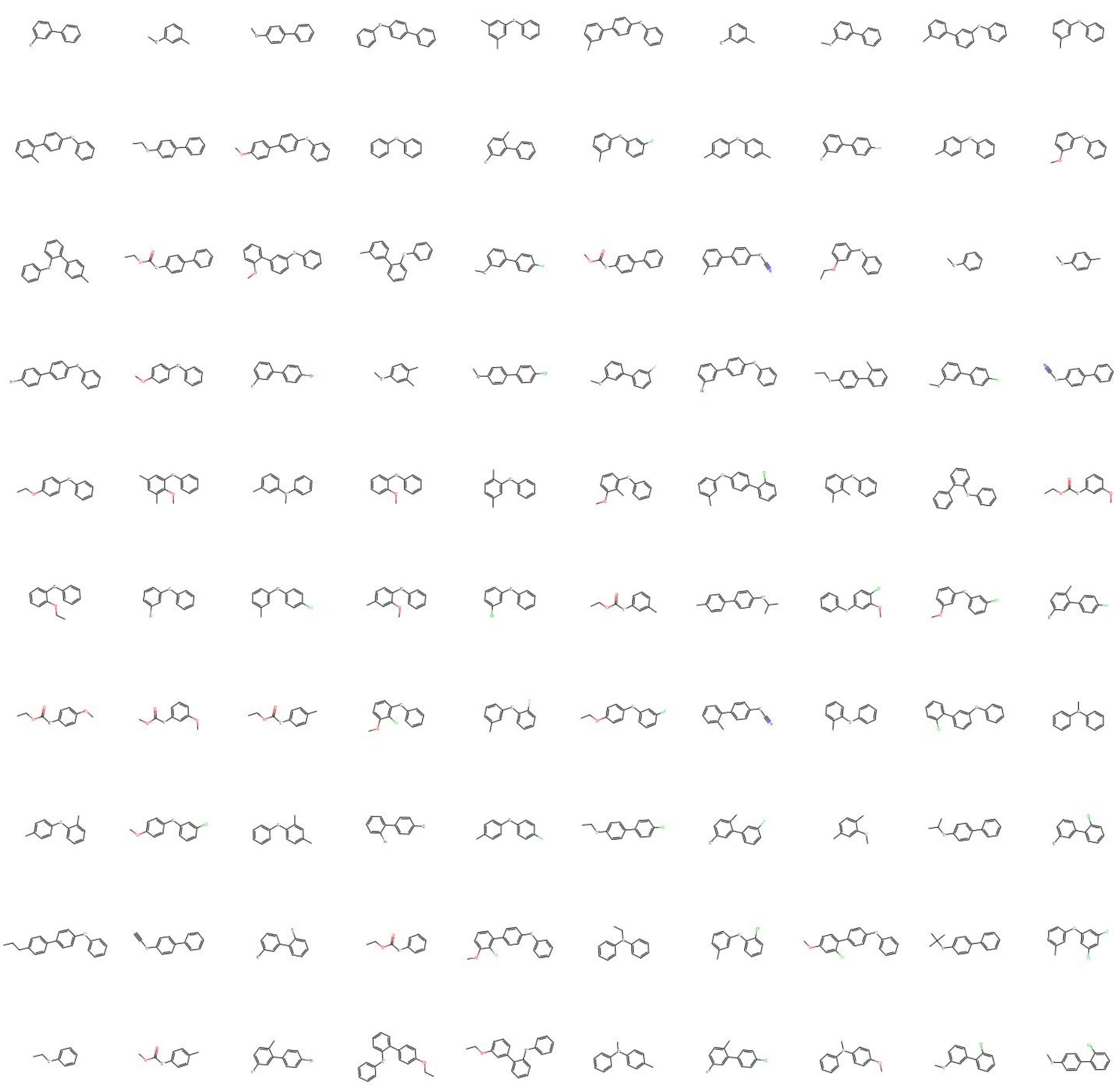

Figure S8. Structures for the top 100 generated novel carbanions ranked by the combined score of log *k* penalized by synthetic accessibility.

Table S1. DFT calculated and ML predicted log *k* values of the 80 carbanions.

Structure	SMILES	ΔΔG$_{calc}$ (kcal/mol)	log k_{calc}	log k_{ML}
**1**	Cc1cc(C)cc([CH-]c2ccccc2)c1	6.86	7.77	6.58
**2**	Cc1cc(C)cc([CH-]C#N)c1	9.17	6.07	6.46
**3**	Cc1cccc([CH-]c2ccccc2)c1	7.73	7.13	6.34
**4**	Cc1cccc([CH-]c2cccc(F)c2)c1	7.75	7.12	6.31
**6**	Cc1cccc([CH-]C#N)c1	9.16	6.08	6.27
**8**	CCOC(=O)[CH-]c1cccc(C)c1	8.88	6.28	6.19
**9**	COC(=O)[CH-]c1cccc(OC)c1	9.99	5.47	6.18
**10**	[CH2-]c1cccc(C)c1	6.56	7.99	6.17
**13**	COC(=O)[CH-]c1ccc(C)cc1	5.33	8.89	6.10
**14**	CCOC(=N)[CH-]c1ccccc1	7.23	7.50	6.09
**15**	CCOC(=O)[CH-]c1cccc(OC)c1	8.45	6.60	6.09
**16**	CCOC(=O)[CH-]c1ccc(C)cc1	8.45	6.60	6.06
**17**	Cc1cccc(-c2cccc([CH-]c3ccccc3)c2)c1	7.70	7.15	6.06
**18**	COc1cccc([CH-]C#N)c1	9.36	5.94	6.04
**19**	Cc1cccc(-c2ccc([CH-]C#N)cc2)c1	9.41	5.90	6.03
**21**	Cc1cccc(-c2ccc([CH-]c3ccccc3)cc2)c1	8.32	6.70	6.02
**22**	CCOC(=O)[CH-]c1ccc(C)c(C)c1	8.62	6.47	6.01
**23**	Cc1ccc([CH-]C#N)cc1	8.84	6.31	6.00
**24**	Cc1cccc([CH-]c2cc(Cl)cc(Cl)c2)c1	8.49	6.57	5.98
**26**	Cc1cccc([C-](C)c2ccccc2)c1	7.59	7.23	5.97
**27**	COc1c(C)cc(C)cc1[CH-]c1ccccc1	8.01	6.92	5.97
**28**	Cc1ccc([CH-]c2ccc(C)cc2)cc1	6.53	8.01	5.95
**30**	COc1cccc([CH-]c2ccccc2)c1	8.05	6.89	5.93
**31**	COC(=O)[CH-]c1ccccc1	6.29	8.19	5.93
**32**	Cc1ccc([CH-]c2ccccc2)cc1	6.59	7.97	5.91
**33**	COc1cc([CH-]C#N)ccc1Cl	9.60	5.76	5.90
**34**	CCOC(=O)[CH-]c1ccccc1	6.80	7.81	5.89
**35**	CCOC(=O)[CH-]c1ccc(OC)cc1	8.10	6.86	5.89
**36**	Brc1cccc([CH-]c2ccccc2)c1	7.77	7.10	5.88
**37**	CCOC(=O)[CH-]c1cccc(Br)c1	9.47	5.85	5.88
**38**	CCOc1cccc([CH-]c2ccccc2)c1	7.53	7.27	5.85
**39**	c1ccc([CH-]c2ccccc2)cc1	7.11	7.58	5.83
**40**	COc1cccc([CH-]c2cccc(Cl)c2)c1	7.71	7.14	5.82
**41**	Cc1cccc([CH-]c2ccccc2)c1C	6.63	7.94	5.82
**42**	N#C[CH-]c1ccccc1	9.25	6.02	5.82
**43**	Cc1cccc([CH-]c2ccccc2F)c1	7.91	7.00	5.81
**45**	Cc1ccc(C)c([CH-]c2ccccc2)c1	6.90	7.74	5.81
**46**	Cc1cccc([CH-]c2ccc(Cl)cc2)c1	7.77	7.10	5.80
**51**	N#C[CH-]c1cccc(F)c1	10.01	5.45	5.78
**52**	COc1cccc([CH-]c2ccccc2)c1C	7.03	7.64	5.78
**54**	COc1cc([CH-]c2ccccc2)ccc1Cl	8.11	6.85	5.76

**55**	N#C[CH-]c1ccc(-c2ccccc2)cc1	**9.81**	**5.61**	**5.76**
**56**	Cc1cccc([C-](C)C#N)c1	**8.26**	**6.74**	**5.76**
**57**	Clc1cccc([CH-]c2ccccc2)c1	**7.85**	**7.04**	**5.75**
**60**	COC(=O)[CH-]c1cccc(Cl)c1	**8.58**	**6.51**	**5.74**
**61**	CC[C-](c1ccccc1)c1ccccc1	**7.56**	**7.25**	**5.74**
**62**	O=C(O)[CH-]c1ccccc1	**7.52**	**7.28**	**5.73**
**63**	COc1cccc([CH-]c2ccccc2)c1Cl	**8.47**	**6.58**	**5.72**
**64**	Cc1ccccc1-c1ccc([CH-]c2ccccc2)cc1	**7.85**	**7.04**	**5.71**
**66**	COC(=O)[CH-]c1ccc(-c2ccccc2)cc1	**7.80**	**7.08**	**5.70**
**67**	Cc1cccc([CH-]c2ccccc2Cl)c1	**8.30**	**6.71**	**5.70**
**68**	Cc1ccccc1-c1ccc([CH-]C#N)cc1	**9.75**	**5.65**	**5.69**
**69**	COc1cc(C)ccc1[CH-]c1ccccc1	**5.46**	**8.79**	**5.68**
**71**	COc1ccc([CH-]c2ccccc2)cc1	**6.37**	**8.12**	**5.68**
**72**	CCOC(=O)[CH-]c1ccc(-c2ccccc2)cc1	**7.60**	**7.22**	**5.67**
**73**	Cc1cccc([CH-]C#N)c1C	**8.70**	**6.42**	**5.67**
**74**	COC(=O)[CH-]c1ccc(F)cc1	**5.44**	**8.80**	**5.66**
**75**	CCOC(=O)[CH-]c1cccc(Cl)c1	**9.68**	**5.70**	**5.66**
**76**	Cc1ccc([C-](C)c2ccccc2)cc1C	**7.44**	**7.34**	**5.65**
**77**	COc1ccccc1[CH-]c1ccccc1	**5.82**	**8.53**	**5.65**
**78**	Cc1ccc([CH-]C#N)c(C)c1	**5.45**	**8.80**	**5.65**
**79**	N#C[CH-]c1cccc(Cl)c1	**10.11**	**5.39**	**5.65**
**80**	CCOc1ccc([CH-]c2cccc(F)c2)cc1	**6.88**	**7.75**	**5.64**
**81**	CCOC(=O)[CH-]c1ccc(OCC)cc1	**6.16**	**8.28**	**5.64**
**82**	CC(=CC#N)[CH-]c1ccccc1	**11.27**	**4.53**	**5.64**
**83**	Cc1ccc([C-](C)c2ccccc2)cc1	**7.23**	**7.50**	**5.63**
**84**	CN(C)C(=O)[CH-]c1ccccc1	**8.16**	**6.81**	**5.62**
**85**	CCCC=C(C)[CH-]c1ccccc1	**7.10**	**7.59**	**5.61**
**86**	COc1ccc([CH-]c2cccc(Cl)c2)cc1	**6.74**	**7.85**	**5.61**
**87**	C[C-](c1ccccc1)c1ccccc1	**7.57**	**7.25**	**5.60**
**88**	CCOc1ccc([CH-]c2ccccc2)cc1	**7.32**	**7.43**	**5.60**
**89**	Cc1ccc([CH-]c2ccc(F)cc2)cc1	**6.50**	**8.03**	**5.60**
**90**	Cc1ccc([CH-]c2ccccc2)c(C)c1	**6.57**	**7.98**	**5.59**
**91**	COc1ccccc1-c1cccc([CH-]c2ccccc2)c1	**6.44**	**8.08**	**5.59**
**92**	Cc1cccc(-c2ccccc2[CH-]c2ccccc2)c1	**6.87**	**7.76**	**5.59**
**93**	COc1ccc([CH-]c2cc(Cl)cc(Cl)c2)cc1	**7.93**	**6.98**	**5.58**
**95**	COc1ccccc1[CH-]C#N	**5.67**	**8.64**	**5.57**
**96**	COc1cccc([C-](C)C#N)c1	**9.18**	**6.07**	**5.57**
**97**	CC=Cc1cccc([CH-]c2ccccc2)c1	**6.94**	**7.70**	**5.56**
**98**	CCOC(=O)[CH-]c1ccc(Br)cc1	**7.13**	**7.57**	**5.56**
